# *Alphaherpesvirinae and Gammaherpesvirinae* glycoprotein L and CMV UL130 originate from chemokines

**DOI:** 10.1186/1743-422X-10-1

**Published:** 2013-01-02

**Authors:** Maja Malkowska, Katarzyna Kokoszynska, Magdalena Dymecka, Leszek Rychlewski, Lucjan S Wyrwicz

**Affiliations:** 1 Laboratory of Bioinformatics and Biostatistics, Maria Sklodowska-Curie Memorial Cancer Center and Institute of Oncology, WK Roentgena 5, 02-781, Warsaw, Poland; 2 BioInfoBank Institute, Limanowskiego 24A, 60-744, Poznań, Poland

**Keywords:** *Herpesviridae*, Glycoprotein L, GL, UL130, Chemokines, HCMV, HSV2, EBV

## Abstract

*Herpesviridae* is a large family of DNA viruses divided into three subfamilies: *Alpha*-, *Beta*- and *Gammaherpesvirinae*. The process of herpesvirus transmission is mediated by a range of proteins, one of which is glycoprotein L (gL). Based on our analysis of the solved structures of HSV2 and EBV gH/gL complexes, we propose that *Alphaherpesvirinae* and *Gammaherpesvirinae* glycoprotein L and *Betaherpesvirinae* UL130 originate from chemokines. Herpes simplex virus type 2 gL and human cytomegalovirus homolog (UL130) adopt a novel C chemokine-like fold, while Epstein-Barr virus gL mimics a CC chemokine structure. Hence, it is possible that gL interface with specific chemokine receptors during the transmission of *Herpesviridae*. We conclude that the further understanding of the function of viral chemokine-like proteins in *Herpesviridae* infection may lead to development of novel prophylactic and therapeutic treatment.

## Background

As a family of dsDNA viruses, Herpesviridae are known to infect vertebrates, including humans, and cause progression of various contagious diseases, such as orofacial herpes (HSV1), genital herpes (HSV2), chickenpox and shingles (VZV), opportunistic infections (CMV), Kaposi’s sarcoma (KSHV) and mononucleosis (EBV) [1]. Members of the *Herpesviridae* family have been classified into three distinct subfamilies: *Alpha-* (HSV1, HSV2, VZV); *Beta-* (CMV, HHV-6, HHV-7); *Gammaherpesvirinae* (EBV, KSHV).

The cell-to-cell transmission of virions is one of limiting steps in spread of infection. The targeting of a specific tissue for infection is based on the recognition of definite entry receptors [2]. The virus used as a model of herpetic infection is HSV1. In its genome, 11 out of 80 genes encode outer membrane glycoproteins. According to previous studies, glycoprotein D (gD), glycoprotein B (gB) and the complex of glycoproteins H and L (gH/gL) are curial proteins for cell-to-cell fusion [3,4]. Although numerous studies on gH/gL biology have been carried out, there is still no unequivocal evidence whether this complex plays role in the recognition of specific cells (tissues) or directly takes part in membrane fusion [5].

The chemokine family has been created based on structural similarity. The characteristics features are the Greek key shape and conserved cysteine residues forming SS-bonds and stabilizing the structure. Chemokine family differs in terms of sequence similarity, which vary from less than 20% to over 90% [6]. Based on the position of conserved cysteines chemokine classification divides them into four sub-families (CC, CXC, C and CX_3_C).

Chemokine mimicry is a common phenomenon among herpesvirus, poxvirus and retrovirus families [7-9]. So far, over 30 chemokine and chemokine receptor mimics have been described. Except for HCMV UL130 and HSV2 gL no other viral proteins have been designated as C type chemokine homologs [10-12].

### Presentation of the hypothesis

In our previous report, based on bioinformatic analysis of protein families, we concluded the possibility of a distant homology between *Herpesviridae* glycoprotein L (gL) and chemokines [12]. We also remarked that human Cytomegalovirus (HCMV) contains an additional gene (UL130) which might encode an orthologous chemokine-like protein. This study was followed by crystallographic determination of the structure of two gH/gL complexes. Chowdary and co-workers crystallographically determined the structure of the HSV2 (Herpes simplex virus 2) gH/gL complex, whilst Matsuura et al. described the crystal structure of an Epstein-Barr virus (EBV) complex [13,14].

Although not noticed in the original reports by Chowdary et al. and Matsuura et al., it is important to note that the topologies of both solved glycoproteins are highly concordant with the homology models [12].

We hypothesize that the betaherpevirus UL130 as well as alpha- and gammaherpesvirus gL proteins exhibit structural similarity with chemokines and may interact with their receptors.

**Figure 1:**
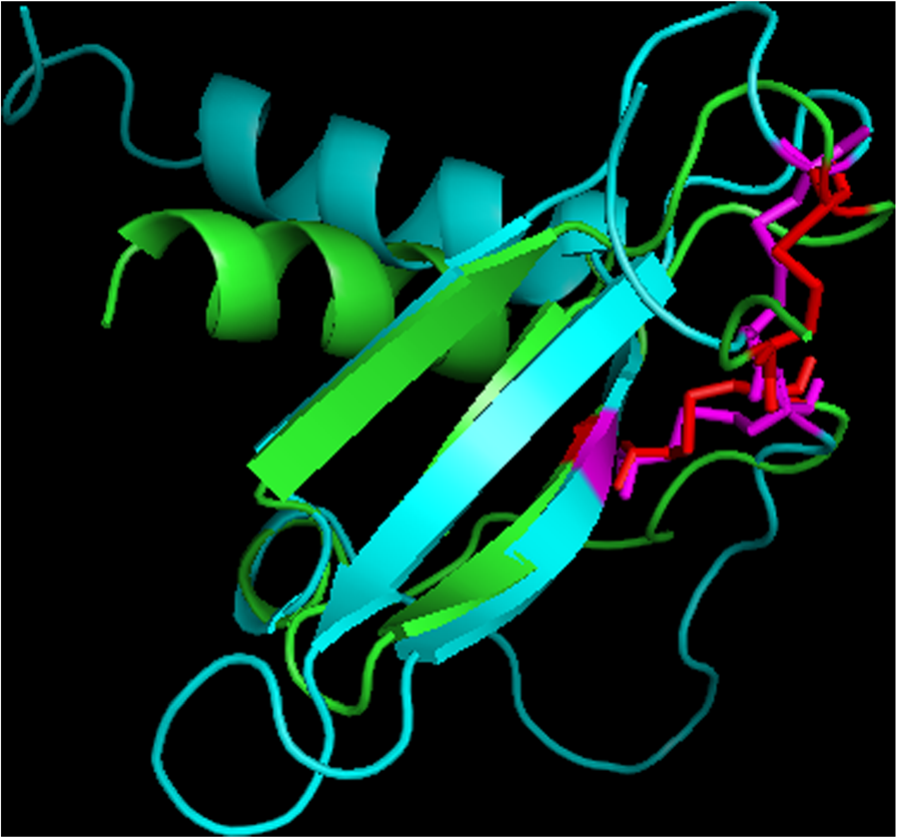
**Superimposed native structures of C-C motif chemokine 5 (green; PDB entry: 1U4L A) and EBV glycoprotein L (cyan; PDB entry 3PHF B).** The cysteine residues are marked in red (C-C motif chemokine 5) and magenta (gL).

**Figure 2:**
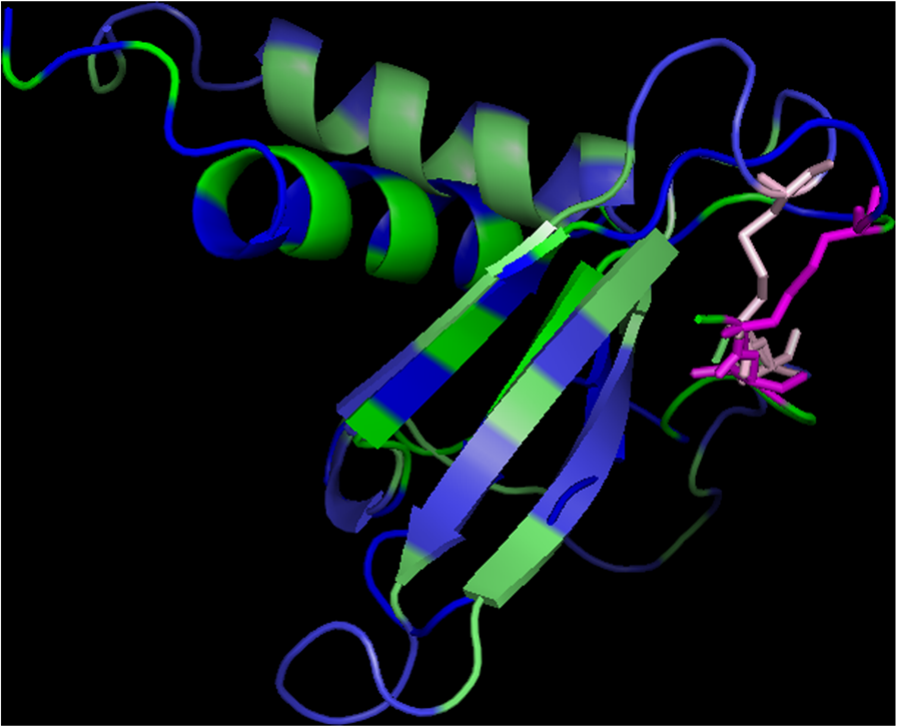
**Superimposed native structures of the CC-type chemokine eotaxin-2 (green; PDB entry: 1EIG A) and EBV glycoprotein L (light-green; PDB entry 3PHF B).** The hydrophobic surfaces are marked in blue (eotaxin-2) and light-blue (gL), cysteine residues in magenta (eotaxin-2) and light-pink (gL).

The structural comparison between EBV gL and CC-type chemokines leaves no doubt that it adopts a CC chemokine-like fold. Figure 1 shows the superimposed structures of a CC-type chemokine (human C-C motif chemokine 5; Protein Data Bank entry: 1U4L_A) and the native structure of EBV glycoprotein L (Protein Data Bank entry 3PHF_B) which exhibit a root mean square deviation (RMSD) for C-alpha atoms of 1.23 Å (analysis performed for atoms forming the curved beta-sheet). The conserved topology is a result of the presence of two cysteine bridges (see Figure 1). Furthermore, the putative interaction surface of EBV’s glycoprotein L with chemokines is also preserved (for the conserved pattern of hydrophobic amino acids see Figure 2). The major difference is a result of the altered angle between the helix and the curved beta-sheet, which is to be expected, since the helix in chemokines is involved in dimerization during receptor recognition, whilst gL lacks the ability to dimerize. A further difference can be seen in the presence of two additional helices at the C-terminus of glycoprotein L – 15 and 6 residues long respectively, separated by a link of only two amino acids.

**Figure 3:**
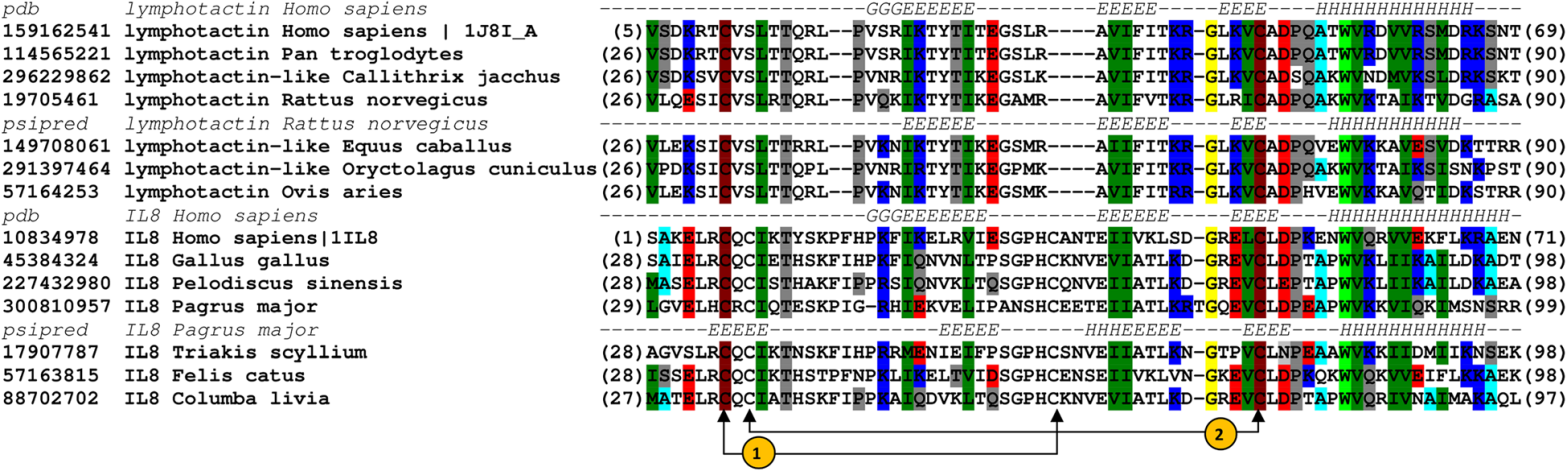
**Multiple sequence alignment of lymphotactin (XCL1) and interleukin-8 (IL-8).** The corresponding sequences were labeled with their GenBank entries (denoted by the GenBank identifier - gi) and organism of origin. The numbers in brackets refer to the positions of the presented sequence fragments. The observed (Protein Data Bank entries 1J8I_A for XCL1 and 1IL8_A for IL-8) and predicted (Psipred) secondary structure elements are coded with letters (H - a-helix, G - 3_10_ helix, E - b-strands). Disulphide bridges in IL-8 has been marked with arrows and numbered.

**Figure 4:**
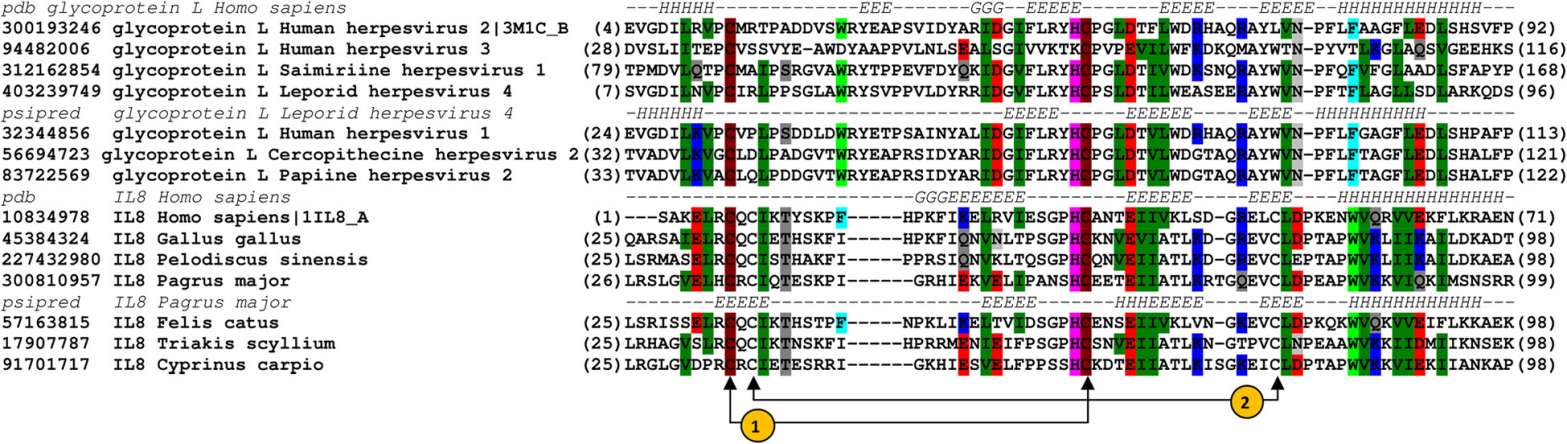
**Multiple sequence alignment of glycoprotein L (found in *Alphaherpesvirinae) *and interleukin-8 (IL-8).** The corresponding sequences were labeled with their GenBank entries (denoted by the GenBank identifier - gi) and organism of origin. The numbers in brackets refer to the positions of the presented sequence fragments. The observed (Protein Data Bank entries 3M1C_B for gL and 1IL8_A for IL-8) and predicted (Psipred) secondary structure elements are coded with letters (H - a-helix, G - 3_10_ helix, E - b-strands). Disulphide bridges in IL-8 has been marked with arrows and numbered.

Comparison of cysteine positions in CXC motif in interleukine-8 (IL-8) and lymphotactin (C type chemokine) has shown the conservation of 1^nd^ and 4^th^ cysteine (Figure 3), whereas in case of IL-8 and gL from HSV2 the 1^st^ and 3^rd^ cysteines are conserved (Figure 4). Despite the non-conserved cysteine position, based on structural similarity, we propose that gL HSV2 shall be classified as C type chemokine-like protein.

**Figure 5:**
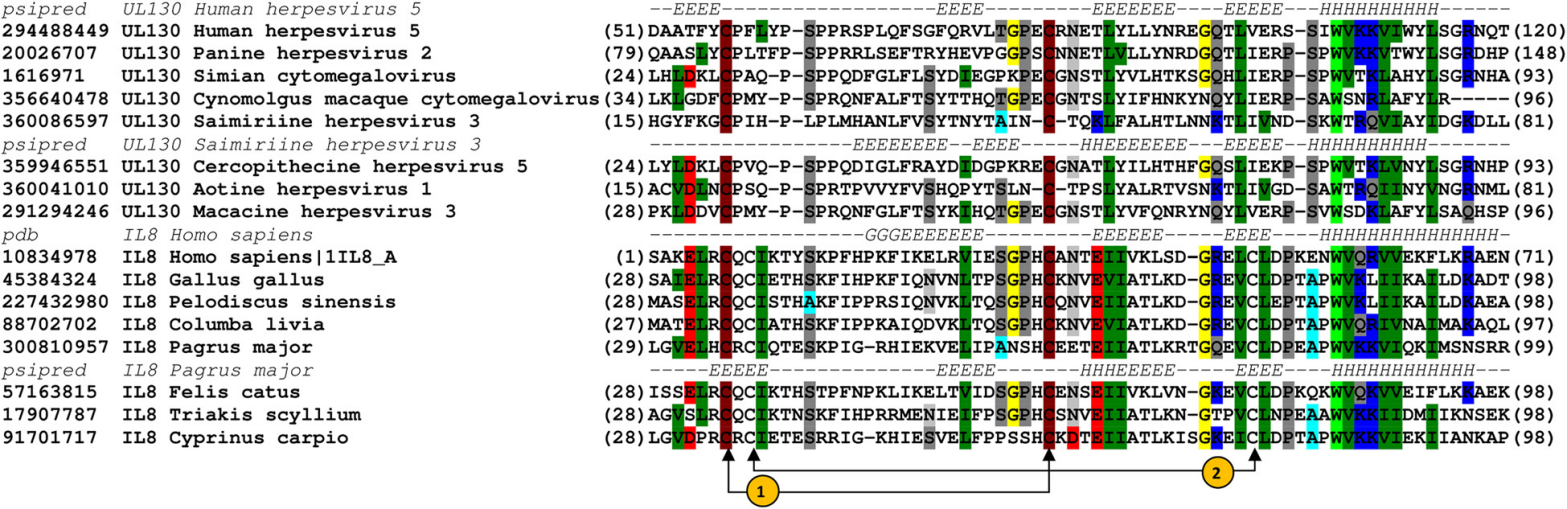
**Multiple sequence alignment of UL130 (found in *Betaherpesvirinae) *and interleukin-8 (IL-8).** The corresponding sequences were labeled with their GenBank entries (denoted by the GenBank identifier - gi) and organism of origin. The numbers in brackets refer to the positions of the presented sequence fragments. The observed (Protein Data Bank entry 1IL8_A for IL-8) and predicted (Psipred) secondary structure elements are coded with letters (H - a-helix, G - 3_10_ helix, E - b-strands). Disulphide bridges in IL-8 has been marked with arrows and numbered.

Due to synteny, rat cytomegalovirus r131 gene encoding a proinflammatory CC chemokine-like protein has been recognized as HCMV UL130 homolog [15,16]. Sequence identity and similarity between r131 and UL130 is 19.1% and 41.1% respectively (calculations were performed with PAM250 scoring matrix for global alignment). The position of conserved cysteines (1^st^ and 3^rd^ cysteine of characteristic CXC type chemokine motif) leads to the assignment of UL130 to C type chemokine subfamily (Figure 5). Despite of the loss of 2^nd^ and 4^th^ cytosine the chemotactic activity might be preserved and requires further investigation as it might explain the mechanism of UL130 functioning in Endothelial Cell infection [17].

Mutagenesis of interleukine-8 showed that perturbation of the cysteine bridge between 2^nd^ and 4^th^ motif element had small effects on its function whereas the modification of the disulfide between 1^st^ and 3^rd^ cysteine dramatically reduced potency [18]. This fact supports our hypothesis that partial conservation of cysteines in UL130 does not necessarily imply a lack of chemotactic activity.

The speculation concerning the co-evolution of the two interacting glycoproteins [14] causes us to presume that the most likely cause of such concordance is that glycoprotein L arose as a result of lateral gene transfer from the host. It is very likely that early in the evolution of *Herpesviridae*, glycoprotein L acted predominantly as a virus encoded chemokine (compare viral chemokines: UL146, UL147, UL152 encoded by HCMV [19]), which later co-evolved with the other membrane viral genes.

In presented analysis we have used a typical homology modeling workbench. The sets of homologs from the NR database (NCBI; National Center for Biotechnology) were created using PSI-BLAST [20] search method and aligned with ClustalW [21]. Secondary structure predictions for selected proteins were calculated using PSIPRED method [22]. Alignment figures were made with the use of BioEdit version 7.1.3 alignment editor [23]. Residue conservation were presented in alignment with the following default for the software scheme: positively charged highlighted in red; negatively charged in blue; hydrophobic in dark green; tryptophan in light green; polar uncharged and proline in gray; histidine in magenta; glycine in yellow; alanine, phenylalanine and tyrosine in cyan; cysteine in maroon. Modeller 9 was used to create homology models [24]. The structures were visualized with PyMOL v. 0.99rc6 which also enables RMSD calculations [25].

### Testing the hypothesis

Very low sequence similarity between various chemokines and gL prevents any phylogenetic studies of the origin of these protein families from being carried out, and we are unable to justify whether the different topology types (CC for *Gammaherpesvirinae*, C for the Alpha group and HCMV) come from a single event in the early divergence of *Herpesviridae*, or rather arose from two independent events of lateral gene transfer.

Discussed here glycoproteins are present in viral envelope and mediate virus entry to host cells. Their mechanism and function as chemokine-like proteins however, remains unknown. There is a potential for further analysis by experimental characterization of specific chemotactic activities of gL and UL130 proteins. Necessary step would be in vivo expression during viral persistence and verification of their release from cells. To establish if these proteins are functional chemokines and which cells are their targets in vivo migration assays shall be performed. Site-directed mutagenesis would help to determine functional domains that are critical for migration. Ligand-binding assays could indicate binding affinity to chemokine receptors.

### Implications of the hypothesis

We conclude that gL could interact with specific cellular chemokine receptors during the invasion of *Herpesviridae*. The results of our study lead to the conclusion that the herpesvirus transmission process can be dependent on gL interaction with specific cellular chemokine receptors. The presented hypothesis might become a basis for creating new vaccine targets as well as binding inhibitors, which will contribute to the development of novel therapeutic and prophylactic strategies. However, all the assumptions require further studies [26,27]. In order to understand the role of gL in infection and answer questions about its exact functioning, further study is required. The analogical role of UL130 in HCMV and its role in infection needs further explanation.

## Competing interests

The authors declare that they have no competing interests.

## Authors’ contributions

MM, KK, LSW participated in the design of the study. MM, KK, MD, LR and LSW carried out the bioinformatics analysis. MM, KK, LSW wrote the manuscript. All authors read and approved the final manuscript.
